# Kasein als seltener Auslöser einer augmentationsfaktorabhängigen Nahrungsmittelallergie ‐ ein Fallbericht: Casein as rare elicitor of food allergy dependent on augmentation factors ‐ A case report

**DOI:** 10.1111/ddg.70275x

**Published:** 2026-11-10

**Authors:** Charlotte Jasmin Kiani, Valentina Faihs, Julia Felicitas Pilz, Claudia Kugler, Tilo Biedermann, Knut Brockow

**Affiliations:** ^1^ Klinik für Dermatologie und Allergologie des Klinikum Rechts der Isar Technische Universität München München Deutschland; ^2^ Odense Research Center for Anaphylaxis (ORCA) Abteilung für Dermatologie und Allergologie Odense Universitätsklinik Odense Dänemark

**Keywords:** Anaphylaxie, FALDA, FDEIA, Kasein, Nahrungsmittelallergie, anaphylaxis, casein, FALDA, FDEIA, food allergy

Sehr geehrte Herausgeber,

Eine 31‐jährige Patientin mit atopischer Dermatitis in der Kindheit sowie milder allergischer Rhinokonjunktivitis auf Gräserpollen stellte sich in unserer Ambulanz mit einer zehnjährigen Anamnese rezidivierender systemischer allergischer Reaktionen im Zusammenhang mit körperlicher Belastung vor. Die Symptome umfassten Kribbeln der Extremitäten, generalisierte Urtikaria, Schwellung im Halsbereich, gastrointestinale Beschwerden sowie in einem Fall Bewusstlosigkeit. Insgesamt traten etwa 16 systemische Episoden auf, überwiegend bei warmen Temperaturen. Zwischen den Reaktionen war körperliche Aktivität beschwerdefrei möglich. Der anfängliche Verdacht auf eine weizenabhängige, augmentationsfaktorabhängige Allergie (Wheat Allergy Dependent on Augmentation Factors; WALDA) führte zu einer strikten weizenfreien Diät über 1,5 Jahre, jedoch ohne klinische Besserung. Die zwei jüngsten Episoden ereigneten sich zwei Monate vor der Vorstellung.

Bei der ersten Episode berichtete die Patientin über den Verzehr eines Salates mit verschiedenen Gemüsesorten, Mozzarella und Sonnenblumenkernen. Zwei Stunden später, während des Joggens, beklagte sie eine generalisierte Urtikaria mit Angioödem, gastrointestinale Symptome und schließlich Bewusstseinsverlust, sodass eine notärztliche Behandlung erforderlich war. Zwei Wochen später kam es im Rahmen eines Infekts der oberen Atemwege zu einer ähnlichen Reaktion während körperlicher Belastung, zwei Stunden nach dem Verzehr von Halloumi, Fenchel, Petersilie und Kichererbsen.

Die Hautpricktestung (Prick‐to‐Prick‐Methode) zeigte eine positive Reaktion auf mehrere Kuhmilchprodukte, darunter Quark (Quaddel 3 mm/Erythem 5 mm), Mozzarella (3/10), ein proteinreiches Milchgetränk (3/10) und Joghurt (5/17) (ergänzende Abbildung S1). Der Pricktest auf Halloumi sowie auf andere konsumierte Nahrungsmittel (Sonnenblumenkerne, Kichererbsen, Petersilie, Fenchel) blieben negativ. Serologisch zeigte sich ein normales Gesamt‐IgE (25 kU/L), jedoch ein erhöhtes spezifisches IgE (sIgE; ImmunoCAP‐Assay, Thermo Fisher Scientific, Uppsala, Schweden) gegen Milch‐Kasein (nBos d 8; 0,61 kU/L). Für Gesamtmilchprotein, α‐Laktalbumin, β‐Laktoglobulin sowie für weitere vor den Reaktionen verzehrte Nahrungsmittel und für Pru p 3, Alpha‐Gal und Omega‐5‐Gliadin wurden keine sIgE nachgewiesen (alle < 0,1 kU/L).

Entsprechend den aktuellen Leitlinien wurde eine orale Provokationstestung (OFC) durchgeführt (Abbildung 1).^1^ Zunächst wurde eine Kombination von Augmentationsfaktoren allein (1 g Acetylsalicylsäure (ASS), 20 mL 95% Alkohol in Tee verdünnt (Braun, Melsungen, Deutschland), 25 Minuten intensives Laufen auf dem Laufband) ohne Symptome toleriert. Auch die Einnahme von 50 g Halloumi (ein vorgekochter Käse mit hitzestabilem Kasein) sowie anschließend 150 g in Kombination mit 1 g ASS wurden beschwerdefrei vertragen. Am folgenden Tag erfolgte die Einnahme von 105 g Halloumi, kombiniert mit 1 g ASS und 20 mL 95% Alkohol, gefolgt von körperlicher Belastung auf dem Laufband. Nach zehn Minuten Laufen entwickelte die Patientin starken Pruritus, eine generalisierte Urtikaria sowie ein Angioödem der Augenlider. Kurz darauf traten Übelkeit, Globusgefühl, Schwindel, Zittern, Hypotonie (89/52 mmHg; initial 128/61 mmHg) und Tachykardie (139/min) auf. Unverzüglich wurde eine antiallergische Therapie mit 300 µg intramuskulärem Adrenalin sowie intravenösem Dimetindenmaleat (8 mg) und Prednisolon (250 mg) eingeleitet, worunter es zu einer vollständigen Rückbildung der Symptome kam. Ein signifikanter Anstieg der Serumtryptase (initial 3,4 µg/L; 30 Minuten nach Reaktion: 12,6 µg/L; 90 Minuten: 18,3 µg/L) bestätigte das Vorliegen einer Anaphylaxie. Damit wurde die Diagnose einer kaseininduzierten, augmentationsfaktorabhängigen Nahrungsmittelallergie (Food Allergy Dependent on Augmenation Factors; FALDA) gestellt. Die Patientin erhielt eine ernährungsmedizinische Beratung und wurde angewiesen, Kuhmilchprodukte in größeren Mengen sowie in Kombination mit Augmentationsfaktoren zu meiden.

**ABBILDUNG 1:**
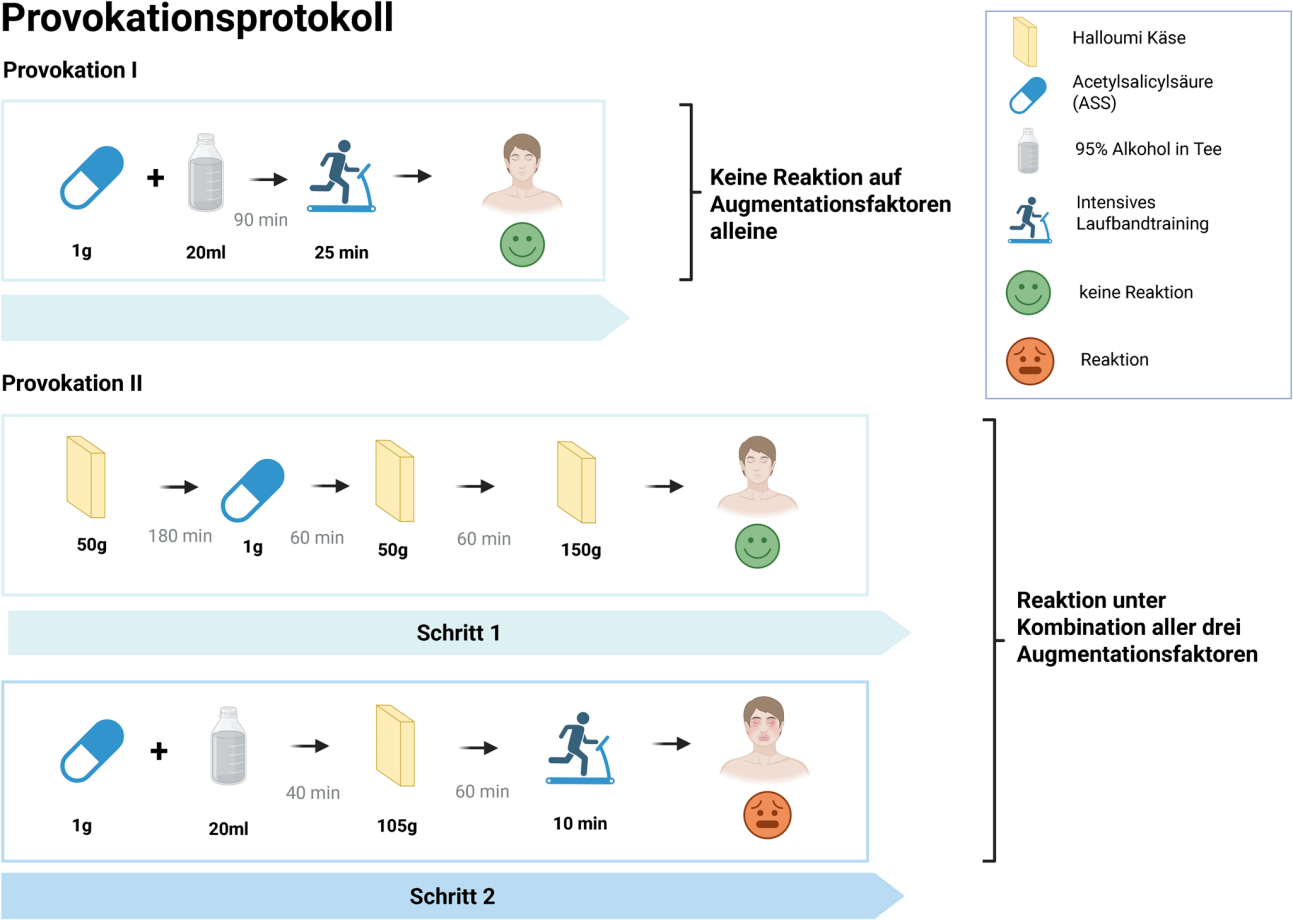
Provokationsprotokoll der oralen Provokationstestung. Provokation I: Testung aller Augmentationsfaktoren ohne Nahrungsmittel zur Ausschlussdiagnostik einer rein belastungsinduzierten Anaphylaxie.  Provokation II: Orale Nahrungsmittelprovokation mit Halloumi zur Verifizierung einer kaseininduzierten, belastungsabhängigen Anaphylaxie. Erstellt mit BioRender. Köberle, M. (2025) https://BioRender.com/4ml4oag.

FALDA stellt eine besondere Form der IgE‐vermittelten Nahrungsmittelallergie dar, die nur in Kombination mit Augmentationsfaktoren wie körperlicher Belastung, der Einnahme nichtsteroidaler Antirheumatika oder Alkoholkonsum ausgelöst wird.^2^, ^3^ Während Kuhmilchproteine bekannte Auslöser der klassischen IgE‐vermittelten Nahrungsmittelallergie darstellen, ist die Rolle bei FALDA bislang nur unzureichend verstanden. Kasein stellt mit etwa 80% den überwiegenden, hitzestabilen Proteinanteil des Kuhmilchproteins dar, während die Molkenproteine die verbleibenden 20% und den hitzelabilen Anteil ausmachen.^4^ Alle Milchprodukte enthalten relevante Mengen an Kasein, allerdings in unterschiedlicher Konzentration abhängig von der Verarbeitung. Unter den in diesem Fall getesteten Produkten weist Käse im Allgemeinen die höchste Kaseinmenge auf. Eine intensive Fermentation und Reifung kann jedoch die Allergenität durch gewisse strukturelle Veränderungen reduzieren.^5^ Dies könnte eine Erklärung dafür sein, warum der Pricktest auf den getesteten Halloumi negativ ausfiel, und warum erst zusätzliche Augmentationsfaktoren in Kombination mit dem Verzehr größerer Mengen Halloumi zu einer klinischen Reaktion führten.

Der erste in der Literatur beschriebene Fall einer kuhmilchinduzierten FALDA wurde 2003 veröffentlicht. Ein Patient litt unter primärer IgE‐vermittelter Kuhmilchallergie in der Kindheit, welche sich im Jugendalter zu einer FALDA wandelte.^6^ Dies unterscheidet sich von der Anamnese unserer Patientin, die von neu auf im Erwachsenenalter eine FALDA entwickelte. Ein erstmaliges Auftreten von Nahrungsmittelallergien im Erwachsenenalter wird insbesondere bei FALDA, vor allem bei der häufigsten Form der WALDA, beschrieben.^7^

Ein weiterer Fall einer kuhmilchinduzierten FALDA zeigte eine Abhängigkeit von der Menstruationsphase der Patientin, was die Komplexität der Diagnosestellung unterstreicht.^8^ Bei unserer Patientin ergab sich anamnestisch keine Abhängigkeit vom Menstruationszyklus. Allerdings war ein einzelner Augmentationsfaktor allein (1 g ASS) nicht ausreichend, um Symptome auszulösen und die orale Provokation nur bei Kombination mehrerer Augmentationsfaktoren positiv.

Bemerkenswert ist, dass bei unserer Patientin die kaseinspezifischen IgE‐Werte nur gering erhöht waren (0,61 kU/L) bei insgesamt normwertigem Gesamt‐IgE (25 kU/L) und der Pricktest auf Halloumi negativ ausfiel. Dennoch kam es sowohl in der oralen Provokationstestung als auch in der berichteten klinischen Anamnese zu eindeutigen und schweren Reaktionen. Dies verdeutlicht, dass Diagnose und Schweregrad nicht zwingend mit dem Sensibilisierungsgrad korrelieren und, dass Augmentationsfaktoren bei einzelnen Patienten die Reaktionsschwelle erheblich senken können.^2^, ^9^ Die orale Provokationstestung bleibt daher essentiell für die Diagnosestellung, um weitere potenziell lebensbedrohliche Ereignisse zu vermeiden und durch Identifikation des auslösenden Triggers die Lebensqualität des Patienten wiederherzustellen.^2^, ^3^, ^10^ Auch bei unserer Patientin wurde die Diagnose erst nach circa zehn Jahren gestellt, was zu mehreren schweren Reaktionen führte.

Zusammenfassend illustriert dieser Fall eine seltene Form der kaseininduzierten FALDA, die durch einen orale Provokationstestung unter Kombination verschiedener Augmentationsfaktoren bestätigt wurde. Der Fall unterstreicht die Notwendigkeit, Kasein nicht nur als Trigger klassischer Kuhmilchallergien, sondern auch im Kontext der FALDA in Betracht zu ziehen. Darüber hinaus verdeutlicht der Fall die diagnostischen Herausforderungen bei FALDA und zeigt, dass eine geringgradige Sensibilisierung in Kombination mit Augmentationsfaktoren klinisch hochrelevant werden kann.

## DANKSAGUNG

Wir danken Martin Köberle für die Erstellung der Abbildungen mit Biorender.com.

Open access Veröffentlichung ermöglicht und organisiert durch Projekt DEAL.

## INTERESSENKONFLIKT

Keiner.

## FÖRDERUNG

Diese Arbeit wurde durch das Bundesministerium für Forschung, Technologie und Raumfahrt (BMTR) im Rahmen des ABROGATE‐Projekts (Förderkennzeichen 01EA2106A) an KB, durch das Clinician Scientist Program TRIAL der Deutschen Gesellschaft für Allergologie und Klinische Immunologie (DGAKI) an VF sowie durch die Deutsche Forschungsgemeinschaft (DFG) im Rahmen des Graduiertenkollegs RTG 2668 (Projekt A3 und A4, Projekt‐ID: 435874434) gefördert.

## Supporting information



Supplementary information

Supplementary information

## References

[1] Santos AF , Riggioni C , Agache I , et al. EAACI‐Leitlinien zur Diagnostik der IgE‐vermittelten Nahrungsmittelallergie. Allergy. 2023;78:3057‐3076.

[2] Srisuwatchari W , Kanchanaphoomi K , Nawiboonwong J , et al. Nahrungsmittelabhängige, belastungsinduzierte Anaphylaxie: Eine eigenständige Form der Nahrungsmittelallergie – aktueller Überblick zu Diagnostik und Therapie. Foods. 2023;12(20):3768.

[3] Faihs V , Kugler C , Schmalhofer V , et al. Weizenabhängige, belastungsinduzierte Anaphylaxie: Subtypen, Diagnostik und Management. J Dtsch Dermatol Ges. 2023;21:1131‐1135. 10.1111/ddg.15162_g 37845057

[4] Giannetti A , Toschi Vespasiani G , Ricci G , et al. Kuhmilchproteinallergie als Modell für Nahrungsmittelallergien. Nutrients. 2021;13(5):1525.

[5] Alessandri C , Sforza S , Palazzo P , et al. Verträglichkeit eines vollständig gereiften Käses bei kuhmilchallergischen Kindern: biochemische, immunchemische und klinische Aspekte. PLoS One. 2012;7:e40945.

[6] García‐Ara MC , Sánchez AV , Martinez MT , et al. Kuhmilchabhängige, belastungsinduzierte Anaphylaxie: Fallbericht eines Patienten mit vorausgegangener Kuhmilchallergie. J Allergy Clin Immunol. 2003;111:647‐648. 10.1067/mai.2003.70 12642852

[7] Kraft M , Dölle‐Bierke S , Renaudin JM , et al. Weizenanaphylaxie bei Erwachsenen unterscheidet sich von Reaktionen auf andere Nahrungsmittel. J Allergy Clin Immunol Pract. 2021;9:2844‐2852. e5.

[8] Bito T , Kanda E , Tanaka M , et al. Kuhmilchabhängige, belastungsinduzierte Anaphylaxie in der prämenstruellen oder ovulatorischen Phase nach kutaner Sensibilisierung. Allergol Int. 2008;57:437‐439.

[9] Brockow K , Kneissl D , Valentini L , et al. Einsatz eines glutenbasierten oralen Provokationstestprotokolls zur Verbesserung der Diagnostik der weizenabhängigen, belastungsinduzierten Anaphylaxie. J Allergy Clin Immunol. 2015;135:977‐984. e4. 10.1016/j.jaci.2014.08.024 25269870

[10] Faihs V , Kugler C , Bent RK , et al. Provokationstest‐bestätigte Diagnose verbessert die Lebensqualität bei kofaktorabhängiger Weizenallergie. Ann Allergy Asthma Immunol. 2023;131:494‐500. e1.

