# Supplementary material for: Kasein als seltener Auslöser einer augmentationsfaktorabhängigen Nahrungsmittelallergie ‐ ein Fallbericht: Casein as rare elicitor of food allergy dependent on augmentation factors ‐ A case report
Source: J Dtsch Dermatol Ges. 2026 Nov 10;24(11):1650–3. [Article in German] doi: 10.1111/ddg.70275x (PMC13749574; doi:10.1111/ddg.70275x)
Supplement: Supplementary file 1 [file DDG-24-1650-s002.pdf]

Prick Test

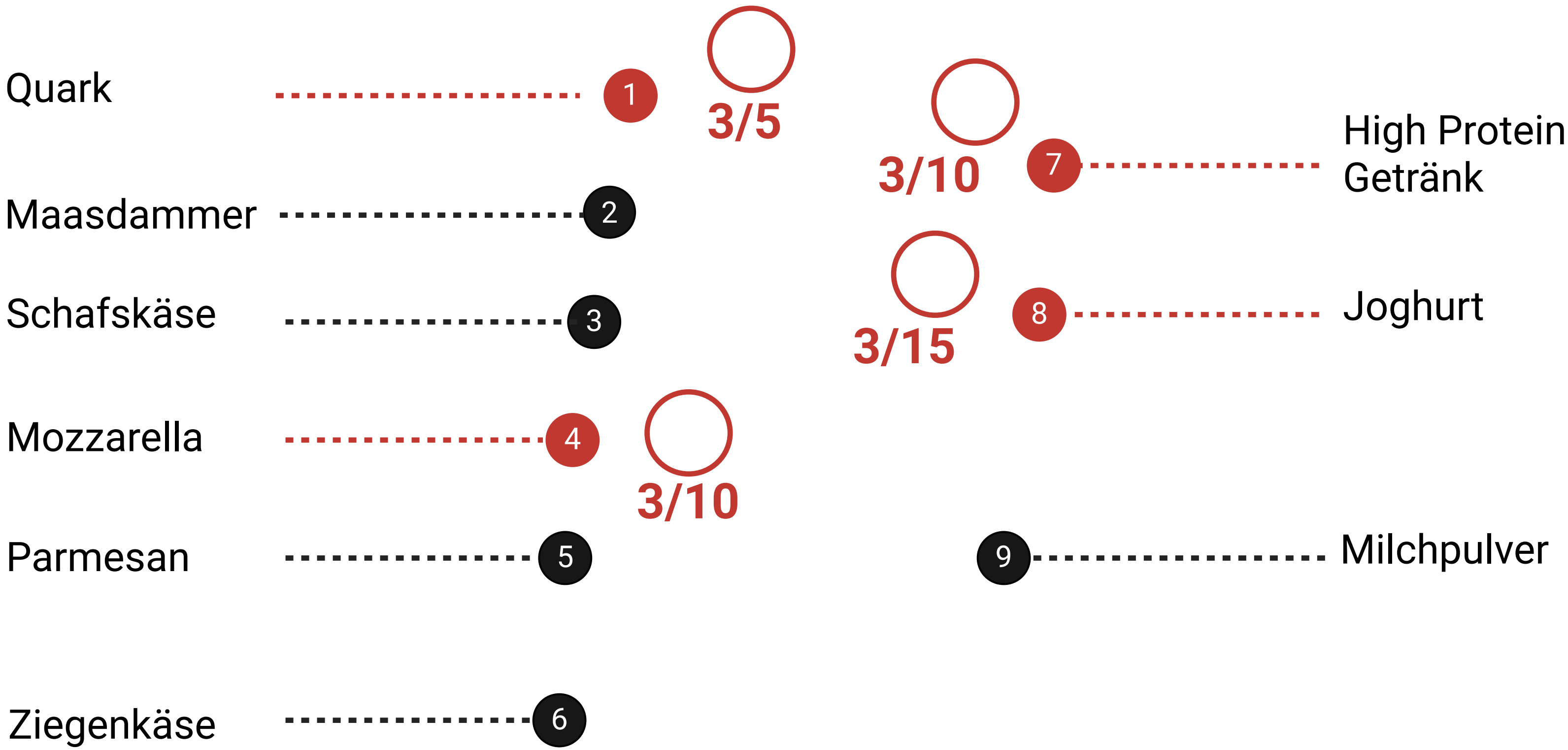

Legende Prick-Test Ergebnisse:
mm Quaddel/mm Erythem
